# Model Selection for Exponential Power Mixture Regression Models

**DOI:** 10.3390/e26050422

**Published:** 2024-05-15

**Authors:** Yunlu Jiang, Jiangchuan Liu, Hang Zou, Xiaowen Huang

**Affiliations:** Department of Statistics and Data Science, College of Economics, Jinan University, Guangzhou 510632, China; tjiangyl@jnu.edu.cn (Y.J.); biubiuyun@outlook.com (J.L.); zouhang19980304@163.com (H.Z.)

**Keywords:** finite mixture of linear regression models, variable selection, exponential power distribution, modified EM algorithm

## Abstract

Finite mixture of linear regression (FMLR) models are among the most exemplary statistical tools to deal with various heterogeneous data. In this paper, we introduce a new procedure to simultaneously determine the number of components and perform variable selection for the different regressions for FMLR models via an exponential power error distribution, which includes normal distributions and Laplace distributions as special cases. Under some regularity conditions, the consistency of order selection and the consistency of variable selection are established, and the asymptotic normality for the estimators of non-zero parameters is investigated. In addition, an efficient modified expectation-maximization (EM) algorithm and a majorization-maximization (MM) algorithm are proposed to implement the proposed optimization problem. Furthermore, we use the numerical simulations to demonstrate the finite sample performance of the proposed methodology. Finally, we apply the proposed approach to analyze a baseball salary data set. Results indicate that our proposed method obtains a smaller BIC value than the existing method.

## 1. Introduction

FMLR models are among the most exemplary statistical tools to deal with various heterogeneous data. Since FMLR models were first introduced by [1,2], they are widely applied in many research fields, e.g., machine learning [3], social sciences [4], and business [5]. For more references to FMLR models, see [6,7,8].

There are two important statistical problems in FMLR models: order selection and variable selection for the different regressions. However, order selection should be the first discussed issue in FMLR models. There exists a lot of literature to deal with this problem. For example, Ref. [9] introduced a penalized likelihood method for mixtures of univariate location distributions. Ref. [10] proposed a penalized likelihood method to select the number of mixing components for the finite multivariate Gaussian mixture models. For variable selection problems for each regression component, Ref. [11] applied subsect selection, REDapproaches such as Akaike information criterion (AIC) and Bayesian information criterion (BIC) to perform a variable selection for each component in a finite mixture of Poisson regression models. To avoid the drawbacks of subsect selection, Ref. [12] introduced a penalized likelihood method for variable selection in FMLR models. Ref. [13] proposed a robust variable selection procedure to estimate and select relevant covariates for FMLR models.

The above-proposed methods do not jointly select the order selection and significant variables in FMLR models. In fact, it is a challenging issue, although some literature exists to solve this problem. Ref. [14] introduced MR-Lasso for FMLR models to simultaneously identify the order selection and significant variables. However, they do not study the large sample properties of the proposed method. Ref. [15] proposed a robust mixture regression estimator via an asymmetric exponential power distribution and [16] studied component selection for exponential power mixture models, while they did not consider the variable selection procedure. Ref. [17] applied the penalized method on the number of components and regression coefficients to conduct model selection for FMLR models, but the error followed a normal distribution. Therefore, the proposed method is very sensitive to the heavy-tailed distribution.

In this paper, motivated by [10,18], we propose a new model selection procedure for the FMLR models via an exponential power distribution, which includes normal distributions and Laplace distributions as special cases. Under some regularity conditions, we investigate the asymptotic properties of the proposed method. In addition, we introduce an expectation-maximization (EM) algorithm [19] and a majorization-maximization (MM) algorithm [20] to solve the proposed optimization problem. The finite sample performance of the proposed method is illustrated via some numerical simulations. Results indicate that the proposed method is more robust to the heavy-tailed distributions than the existing method.

The rest of this paper is organized as follows. In Section 2, we present the finite mixture of regression models with an exponential power distribution and a penalized likelihood-based model selection approach. The asymptotic properties of the resulting estimates are investigated. In Section 3, a modified EM algorithm and an MM algorithm are developed to maximize the penalized likelihood. In Section 4, we propose a data-driven procedure to select the tuning parameters. In Section 5, simulation studies are conducted to evaluate the finite sample performance of the proposed method. In Section 6, a real data set is analyzed to compare the proposed test with some existing methods. We conclude with some remarks in Section 7. Technical conditions and proofs are given in the Appendix A.

## 2. Methodology

The density function of an exponential power (EP) distribution is defined as follows:

$$\begin{array}{c} f_{p}\left(x;0,\sigma \right)=\frac{p}{\Gamma \left(\frac{1}{p}\right)2^{1+\frac{1}{p}}\sigma }\exp\left(-\frac{1}{2}{\left|\frac{x}{\sigma }\right|}^{p}\right), \end{array}$$

where p>0, σ>0 is the scale parameter, and Γ(·) is the Gamma function. When 0<p<2, the EP distribution is heavy-tailed, which indicates that it can provide protection against outliers. The EP density function is a flexible and general density function class, and includes some important statistical density functions as its special cases, e.g., Gaussian density function (p=2), and Laplace density function (p=1). Meanwhile, the EP distribution has a wide range of applications, particularly in the area of business applications [21].

Based on the EP density function, we study the FMLR models. Let *Z* be a latent class variable with $P\left(Z=j|x\right)=\pi_{j}$ for j=1,2,⋯,m, where X is a *p*-dimensional vector. Given Z=j, suppose that the response *Y* depends on X in a linear way

$$Y=X^{T}\beta_{j}+\epsilon_{j},$$

where $\beta_{j}$ is a *p*-dimensional vector, and $\epsilon_{j}$ is a random error with an EP density function $f_{p_{j}}\left(x;0,\sigma_{j}\right)$. Then the conditional density of *Y* given X can be written as

(1) $$f\left(y|x\right)=\sum_{j=1}^{m}\pi_{j}f_{p_{j}}\left(Y-X^{T}\beta_{j};0,\sigma_{j}\right).$$

Let $\left\{\left(X_{1},Y_{1}\right),\cdots ,\left(X_{n},Y_{n}\right)\right\}$ be a random sample from (1). Then, the log-likelihood function for observations $\left\{\left(X_{1},Y_{1}\right),\cdots ,\left(X_{n},Y_{n}\right)\right\}$ is given by

$$Q_{n}\left(\theta \right)=\sum_{i=1}^{n}\log\left[\sum_{j=1}^{m}\pi_{j}f_{p_{j}}\left(Y_{i}-X_{i}^{T}\beta_{j};0,\sigma_{j}\right)\right],$$

where $\theta =(\beta_{11},\cdots ,\beta_{1p},\cdots ,\beta_{m1},\cdots ,\beta_{mp},\sigma_{1},\cdots ,\sigma_{m},p_{1},\cdots ,p_{m},\pi_{1},\cdots ,\pi_{m-1})$.

To deal with the model selection problem, according to [10], we consider the following objective function,

(2) $${\tilde{Q}}_{n}\left(\theta \right)=Q_{n}\left(\theta \right)-P_{n}^{1}\left(\theta \right)-P_{n}^{2}\left(\theta \right)$$

with the penalty function

$$P_{n}^{1}\left(\theta \right)=n\sum_{j=1}^{m}\left\{\sum_{t=1}^{p}p_{\lambda_{1}}\left(\right|\beta_{jt}\left|\right)\right\},$$

$$P_{n}^{2}\left(\theta \right)=n\lambda_{2}\sum_{j=1}^{m}\left[\log\left(\epsilon +p_{\lambda_{2}}\left(\pi_{j}\right)\right)-\log\left(\epsilon \right)\right],$$

where $p_{\lambda }\left(\cdot \right)$ is a non-negative and non-decreasing function, and $\lambda_{1}>0$ and $\lambda_{2}>0$ are two penalized parameters. Thus, we can obtain the estimators ${\hat{\theta }}_{n}$ of θ as follows

(3) $${\hat{\theta }}_{n}=\arg\max_{\theta }{\tilde{Q}}_{n}\left(\theta \right).$$

To derive some theoretical properties of the estimators ${\hat{\theta }}_{n}$, we first define

$$a_{n}=\max_{j,t}\left\{p_{\lambda_{1}}^{\prime }\left(\beta_{jt}^{0}\right)/\sqrt{n},p_{\lambda_{2}}^{\prime }\left(\pi_{j}^{0}\right)/\sqrt{n}:\beta_{jt}^{0}\neq 0,\pi_{j}^{0}\neq 0\right\},$$

$$b_{n}=\max_{j,t}\left\{p_{\lambda_{1}}^{\prime \prime }\left(\beta_{jt}^{0}\right)/n,p_{\lambda_{2}}^{\prime \prime }\left(\pi_{j}^{0}\right)/n:\beta_{jt}^{0}\neq 0,\pi_{j}^{0}\neq 0\right\},$$

where $p_{\lambda }^{\prime }\left(h\right)$ and $p_{\lambda }^{\prime \prime }\left(h\right)$ are the first and second derivatives of the function $p_{\lambda }\left(h\right)$ with respect to *h*. To establish the asymptotic properties of the proposed estimators, we assume the following regularity conditions:

(C1) For any λ, $p_{\lambda }\left(0\right)=0$, and $p_{\lambda }\left(\cdot \right)$ is non-negative and symmetric. Furthermore, it is non-decreasing and twice differentiable in (0,∞) with at most a few exceptions.
(C2) As n→∞, $b_{n}=o\left(1\right)$.
(C3) $$\begin{array}{c} \lim_{n\to \infty }\underset{0<h\leq n^{-1/2}\log n}{\inf}\sqrt{n}p_{\lambda }^{\prime }\left(h\right)=\infty . \end{array}$$
(C4) The joint density f(z,θ) of Z=(X,Y) have the third partial derivatives with respect to θ for almost all z.
(C5) For each $\theta_{0}$, there exists $R_{1}\left(z\right)$ and $R_{2}\left(z\right)$ such that for θ in a neighborhood $N(\theta_{0})$ of $\theta_{0}$,
  $$\begin{array}{c} \left|\frac{\partial f(z;\theta )}{\partial \theta_{i}}\right|\leq R_{1}\left(z\right),\left|\frac{\partial^{2}f\left(z;\theta \right)}{\partial \theta_{i}\partial \theta_{j}}\right|\leq R_{1}\left(z\right),\left|\frac{\partial^{3}f\left(z;\theta \right)}{\partial \theta_{i}\partial \theta_{j}\partial \theta_{k}}\right|\leq R_{2}\left(z\right), \end{array}$$
  where $\theta_{0}$ is the true parameter, $R_{1}\left(z\right)$ and $R_{2}\left(z\right)$ satisfy $\int R_{1}\left(z\right)dz<\infty$, and $\int R_{2}\left(z\right)f\left(z;\theta \right)dz<\infty$.
(C6) The Fisher information matrix Q(θ) is finite and positive definite at $\theta =\theta_{0}$, where Q(θ) is defined as follows,
  $$\begin{array}{c} Q\left(\theta \right)=E\left\{\left[\frac{\partial }{\partial \theta }\log\left(f\left(Z;\theta \right)\right)\right]{\left[\frac{\partial }{\partial \theta }\log\left(f\left(Z;\theta \right)\right)\right]}^{T}\right\}. \end{array}$$
(C7) $p_{j}>1,j=1,\cdots ,m$.
(C8) $c_{1}\leq \sigma_{j}^{2}\leq c_{2},∥\beta_{j}∥\leq c_{3},j=1,\cdots ,m$, where $c_{1}$ is some positive constant, $c_{2}$ and $c_{3}$ are some large constants.

**Remark** **1.** *Conditions C1–C3 are the assumption about the penalty function, and assure that the variable selection of the proposed estimators is consistent. The similar conditions are also used in [22]. Condition (C5) ensures that the main term dominates the remainder in the Taylor expansion. Conditions (C4)–(C6) are used in [17]. Condition (C7) ensures the concavity of the likelihood function since the log likelihood function of random sample from EP distribution is concave if p>1. Condition (C8) ensures the compactness of parameter space. Conditions (C7) and (C8) are similarly applied in Wang and Feng [16].*

In the following, we have two theorems with proofs given in the Appendix A.

**Theorem** **1.** *Under the conditions (C1), (C2), (C4)–(C8), and if $\sqrt{n}\min\left\{\lambda_{1},\lambda_{2}\right\}\to \infty$, and $\min\{\lambda_{1},\lambda_{2}\}\to 0$, then there exists a local maximizer ${\hat{\theta }}_{n}$ of the penalized log-likelihood function (2) such that*

$$\begin{array}{c} \left|\left|{\hat{\theta }}_{n}-\theta_{0}\right|\right|=O_{p}\left(n^{-1/2}\right). \end{array}$$

**Theorem** **2.** *Under the conditions (C1)–(C8), and if $\sqrt{n}\min\left\{\lambda_{1},\lambda_{2}\right\}\to \infty$, and $\min\{\lambda_{1},\lambda_{2}\}\to 0$. Then, for any $\sqrt{n}$-consistent estimator ${\hat{\theta }}_{n}$ of θ, we have*

(a) *Sparsity: $P\{{\hat{\pi }}_{k}=0\}\to 1$ as n→∞, where $k=m_{0}+1,\cdots ,m$.*
(b) *Sparsity: $P\{{\hat{\beta }}_{kj}=0\}\to 1$ as n→∞, where $k=1,\cdots ,m_{0}$ and $j=1,\cdots ,t_{k}$.*
(c) *Asymptotic normality:*
$$\begin{array}{c} \sqrt{n}\left\{\left[Q_{1}\left(\theta_{01}\right)-\frac{P_{n}^{1^{\prime \prime }}\left(\theta_{01}\right)}{n}-\frac{P_{n}^{2^{\prime \prime }}\left(\theta_{01}\right)}{n}\right]\left({\hat{\theta }}_{n1}-\theta_{01}\right)+\frac{P_{n}^{1^{\prime }}\left(\theta_{01}\right)}{n}+\frac{P_{n}^{2^{\prime }}\left(\theta_{01}\right)}{n}\right\}\overset{D}{\to }N\left(0,Q_{1}\left(\theta_{01}\right)\right), \end{array}$$
  *where $m_{0}$ is the number of true non-zero mixing weights, $\theta_{01}$ and $Q_{1}\left(\theta_{01}\right)$ are the true parameter and the corresponding Fisher information when all zero effects are removed, respectively.*

## 3. Algorithm

In this section, we apply a modified EM algorithm and an MM algorithm to solve the proposed optimization problem (3). Let $z_{ij}$ be the indicator variables that show if the *i*-th observation arises from the *j*-th component as missing data, and $p_{ij}$ is the posterior probability that the *i*-th observation belongs to the *j*-th component. Therefore, the expected complete-data log-likelihood function is given as follows:

$$\sum_{i=1}^{n}\sum_{j=1}^{m}z_{ij}\log\left[\pi_{j}f_{p_{j}}\left(Y_{i}-X_{i}^{T}\beta_{j};0,\sigma_{j}\right)\right].$$

Then, the objective function (2) is rewritten as

(4) $$\sum_{i=1}^{n}\sum_{j=1}^{m}p_{ij}\log\left[\pi_{j}f_{p_{j}}\left(Y_{i}-X_{i}^{T}\beta_{j};0,\sigma_{j}\right)\right]-P_{n}^{1}\left(\theta \right)-P_{n}^{2}\left(\theta \right).$$

Next, we apply a modified EM algorithm to maximize the objective function (4). The detailed procedure is given as follows:

**Step 1** Given the *l*-th approximation

$${\hat{\theta }}^{\left(l\right)}=\left({\hat{\beta }}_{11}^{\left(l\right)},\cdots ,{\hat{\beta }}_{1p}^{\left(l\right)},\cdots ,{\hat{\beta }}_{m1}^{\left(l\right)},\cdots ,{\hat{\beta }}_{mp}^{\left(l\right)},{\hat{\sigma }}_{1}^{\left(l\right)},\cdots ,{\hat{\sigma }}_{m}^{\left(l\right)},{\hat{p}}_{1}^{\left(l\right)},\cdots ,{\hat{p}}_{m}^{\left(l\right)},{\hat{\pi }}_{1}^{\left(l\right)},\cdots ,{\hat{\pi }}_{m-1}^{\left(l\right)}\right),$$

we can calculate the classification probabilities:

$${\hat{p}}_{ij}^{\left(l+1\right)}=\frac{{\hat{\pi }}_{j}^{\left(l\right)}f_{{\hat{p}}_{j}^{\left(l\right)}}\left(Y_{i}-X_{i}^{T}{\hat{\beta }}_{j}^{\left(l\right)};0,{\hat{\sigma }}_{j}^{\left(l\right)}\right)}{\sum_{j=1}^{m}{\hat{\pi }}_{j}^{\left(l\right)}f_{{\hat{p}}_{j}^{\left(l\right)}}\left(Y_{i}-X_{i}^{T}{\hat{\beta }}_{j}^{\left(l\right)};0,{\hat{\sigma }}_{j}^{\left(l\right)}\right)}.$$

**Step 2** We first update $\left\{\pi_{1},\cdots ,\pi_{m}\right\}$. We use a Lagrange multiplier δ to take into account for the constraint $\sum_{j=1}^{m}\pi_{j}=1$, then we have

(5) $$\frac{\partial }{\partial \pi_{j}}\left\{\sum_{i=1}^{n}\sum_{j=1}^{m}{\hat{p}}_{ij}^{\left(l+1\right)}\log\left(\pi_{j}\right)-n\lambda_{2}\sum_{j=1}^{m}\left[\log\left(\epsilon +p_{\lambda_{2}}\left(\pi_{j}\right)\right)\right]-\delta \left(\sum_{j=1}^{m}\pi_{j}-1\right)\right\}=0.$$

In (5), we apply the local linear approximation [23] to $\log\left(\epsilon +p_{\lambda_{2}}\left(\pi_{j}\right)\right)$,

$$\log\left(\epsilon +p_{\lambda_{2}}\left(\pi_{j}\right)\right)\approx \log\left(\epsilon +p_{\lambda_{2}}\left({\hat{\pi }}_{j}^{\left(l\right)}\right)\right)+\frac{p_{\lambda_{2}}^{\prime }\left({\hat{\pi }}_{j}^{\left(l\right)}\right)}{\epsilon +p_{\lambda_{2}}\left({\hat{\pi }}_{j}^{\left(l\right)}\right)}\left(\pi_{j}-{\hat{\pi }}_{j}^{\left(l\right)}\right).$$

Then, $\pi_{j}$ can be updated by straightforward calculations,

$${\hat{\pi }}_{j}^{\left(l+1\right)}=\frac{1}{D_{j}}\sum_{i=1}^{n}{\hat{p}}_{ij}^{\left(l+1\right)},$$

where

$$D_{j}=n\left[1-\lambda_{2}\sum_{j=1}^{m}\frac{{\hat{\pi }}_{j}^{\left(l\right)}p_{\lambda_{2}}^{\prime }\left({\hat{\pi }}_{j}^{\left(l\right)}\right)}{\epsilon +p_{\lambda_{2}}\left({\hat{\pi }}_{j}^{\left(l\right)}\right)}+\lambda_{2}\frac{p_{\lambda_{2}}^{\prime }\left({\hat{\pi }}_{j}^{\left(l\right)}\right)}{\epsilon +p_{\lambda_{2}}\left({\hat{\pi }}_{j}^{\left(l\right)}\right)}\right].$$

Next, we update $\left\{\beta_{11},\cdots ,\beta_{1p},\cdots ,\beta_{m1},\cdots ,\beta_{mp},\sigma_{1},\cdots ,\sigma_{m},p_{1},\cdots ,p_{m}\right\}$ by maximizing the following objective function,

$$\begin{array}{c} \sum_{i=1}^{n}\sum_{j=1}^{m}{\hat{p}}_{ij}^{\left(l+1\right)}\log\left[{\hat{\pi }}_{j}^{\left(l+1\right)}f_{p_{j}}\left(Y_{i}-X_{i}^{T}\beta_{j};0,\sigma_{j}\right)\right]-n\sum_{j=1}^{m}\left\{\sum_{t=1}^{p}p_{\lambda_{1}}\left(\right|\beta_{jt}\left|\right)\right\}. \end{array}$$

We first update $\left\{\sigma_{1},\cdots ,\sigma_{m}\right\}$. For each $\sigma_{j},j=1,2,\cdots ,m$, we only need to maximize

$$\sum_{i=1}^{n}{\hat{p}}_{ij}^{\left(l+1\right)}\left(-\log\left(\sigma_{j}\right)-\frac{1}{2}{\left|\frac{Y_{i}-X_{i}^{T}{\hat{\beta }}_{j}^{\left(l\right)}}{\sigma_{j}}\right|}^{{\hat{p}}_{j}^{\left(l\right)}}\right).$$

Then, the resulting estimator is given as follows:

$${\hat{\sigma }}_{j}^{\left(l+1\right)}=\frac{\sum_{i=1}^{n}{\hat{p}}_{ij}^{\left(l+1\right)}{\left|Y_{i}-X_{i}{\hat{\beta }}_{j}^{\left(l\right)}\right|}^{{\hat{p}}_{j}^{\left(l\right)}}}{2\sum_{i=1}^{n}{\hat{p}}_{ij}^{\left(l+1\right)}}.$$

Next, we update $\left\{p_{1},\cdots ,p_{m}\right\}$. For each $p_{j},j=1,2\cdots ,m$, according to the condition (C7), we have

$${\hat{p}}_{j}^{\left(l+1\right)}=\underset{p_{j}>1}{\arg\max}\sum_{i=1}^{n}{\hat{p}}_{ij}^{\left(l+1\right)}\left\{\log\left(p_{j}\right)-log(\Gamma \left(\frac{1}{p_{j}}\right)-\left(1+\frac{1}{p_{j}}\right)\log\left(2\right)-\frac{1}{2}{\left|\frac{Y_{i}-X_{i}^{T}\beta_{j}^{\left(l\right)}}{{\hat{\sigma }}_{j}^{\left(l+1\right)}}\right|}^{p_{j}}\right\}.$$

Finally, we update $\left\{\beta_{1},\cdots ,\beta_{m}\right\}$. By ignoring some terms which do not involve in $\beta_{j}$, we have

$$L\left(\beta_{j}\right)=-\sum_{i=1}^{n}{\hat{p}}_{ij}^{\left(l+1\right)}\frac{1}{2{\hat{\sigma }}_{j}^{\left(l+1\right)}}|Y_{i}-X_{i}^{T}\beta_{j}{|}^{{\hat{p}}_{j}^{\left(l+1\right)}}-n\sum_{t=1}^{p}p_{\lambda_{1}}\left(\right|\beta_{jt}\left|\right).$$

By using a MM algorithm for $L(\beta_{j})$’s the first term, we have

$$\begin{array}{c} {\left\{{\left(Y_{i}-X_{i}^{T}\beta_{j}\right)}^{T}\left(Y_{i}-X_{i}^{T}\beta_{j}\right)\right\}}^{\frac{{\hat{p}}_{j}^{\left(l+1\right)}}{2}}\leq {\left\{{\left(Y_{i}-X_{i}^{T}{\hat{\beta }}_{j}^{\left(l\right)}\right)}^{T}\left(Y_{i}-X_{i}^{T}{\hat{\beta }}_{j}^{\left(l\right)}\right)\right\}}^{\frac{{\hat{p}}_{j}^{\left(l+1\right)}}{2}}\\ +\frac{{\hat{p}}_{j}^{\left(l+1\right)}}{2}{\left\{{\left(Y_{i}-X_{i}^{T}{\hat{\beta }}_{j}^{\left(l\right)}\right)}^{T}\left(Y_{i}-X_{i}^{T}{\hat{\beta }}_{j}^{\left(l\right)}\right)\right\}}^{\frac{{\hat{p}}_{j}^{\left(l+1\right)}}{2}-1}\left\{{\left(Y_{i}-X_{i}^{T}\beta_{j}\right)}^{T}\left(Y_{i}-X_{i}^{T}\beta_{j}\right)-{\left(Y_{i}-X_{i}^{T}{\hat{\beta }}_{j}^{\left(l\right)}\right)}^{T}\left(Y_{i}-X_{i}^{T}{\hat{\beta }}_{j}^{\left(l\right)}\right)\right\}. \end{array}$$

For $p_{\lambda_{1}}\left(\right|\beta_{jt}\left|\right)$, we apply a local quadratic approximation [22], then we have

$$p_{\lambda_{1}}\left(\right|\beta_{jt}\left|\right)\approx p_{\lambda_{1}}\left(\right|{\hat{\beta }}_{jt}^{\left(l\right)}\left|\right)+\frac{p_{\lambda_{1}}^{\prime }\left(\right|{\hat{\beta }}_{jt}^{\left(l\right)}\left|\right)}{2|{\hat{\beta }}_{jt}^{\left(l\right)}|}\left(\beta_{jt}^{2}-{\hat{\beta }}_{jt}^{(l)2}\right).$$

Thus, for each $\beta_{j},j=1,2,\cdots ,m$, we only need to solve the following minimization problem

$${\hat{\beta }}_{j}^{\left(l+1\right)}=\underset{\beta_{j}}{\arg\min}\left[\sum_{i=1}^{n}{\hat{p}}_{ij}^{\left(l+1\right)}\frac{1}{2{\hat{\sigma }}_{j}^{\left(l+1\right)}}{\hat{w}}_{ij}^{\left(l\right)}{\left(Y_{i}-X_{i}^{T}\beta_{j}\right)}^{T}\left(Y_{i}-X_{i}^{T}\beta_{j}\right)+n\sum_{t=1}^{p}\beta_{jt}^{2}\frac{p_{\lambda_{1}}^{\prime }\left(\right|{\hat{\beta }}_{jt}^{\left(l\right)}\left|\right)}{2|{\hat{\beta }}_{jt}^{\left(l\right)}|}\right],$$

where ${\hat{w}}_{ij}^{\left(l\right)}=\frac{{\hat{p}}_{j}^{\left(l+1\right)}}{2}{\left\{{\left(Y_{i}-X_{i}^{T}{\hat{\beta }}_{j}^{\left(l\right)}\right)}^{T}\left(Y_{i}-X_{i}^{T}{\hat{\beta }}_{j}^{\left(l\right)}\right)\right\}}^{\frac{{\hat{p}}_{j}^{\left(l+1\right)}}{2}-1}$.

Thus, we can update $\beta_{j}$ as follows

$${\hat{\beta }}_{j}^{\left(l+1\right)}={\left(XBX^{T}+A\right)}^{-1}XBY,$$

where

$$\begin{array}{ccc} A & = & n*diag\left\{\frac{p_{\lambda_{1}}^{\prime }\left(\right|{\hat{\beta }}_{j1}^{\left(l\right)}\left|\right)}{2\left|{\hat{\beta }}_{j1}^{\left(l\right)}\right|},\cdots ,\frac{p_{\lambda_{1}}^{\prime }\left(\right|{\hat{\beta }}_{jp}^{\left(l\right)}\left|\right)}{2\left|{\hat{\beta }}_{jp}^{\left(l\right)}\right|}\right\},\\ B & = & diag\left\{{\hat{p}}_{1j}^{\left(l+1\right)}\frac{1}{2{\hat{\sigma }}_{j}^{\left(l+1\right)}}{\hat{w}}_{1j}^{\left(l\right)},{\hat{p}}_{2j}^{\left(l+1\right)}\frac{1}{2{\hat{\sigma }}_{j}^{\left(l+1\right)}}{\hat{w}}_{2j}^{\left(l\right)},\cdots ,{\hat{p}}_{nj}^{\left(l+1\right)}\frac{1}{2{\hat{\sigma }}_{j}^{\left(l+1\right)}}{\hat{w}}_{nj}^{\left(l\right)}\right\}. \end{array}$$

**Step 3** Repeat **Step 1**, and **Step 2** until convergence.

## 4. Choice of the Tuning Parameters

The selection of tuning parameters is a vital part in the order selection and variable selection procedure. In order to guarantee that a true model can be chosen correctly, we should select the proper tuning parameters $\lambda_{1}$ and $\lambda_{2}$ in the process of practice. There are lots of methods to select $\lambda_{1}$ and $\lambda_{2}$, such as cross-validation (CV), generalized cross-validation (GCV), AIC, and BIC.

As suggested in [24], we introduce a data-driven procedure to choose the tuning parameters $\lambda_{1}$ and $\lambda_{2}$ by minimizing the following modified Bayesian information criterion,

(6) $$MBIC\left(\lambda_{1},\lambda_{2}\right)=-2\sum_{i=1}^{n}\log\left\{\sum_{j=1}^{\hat{m}}{\hat{\pi }}_{j}f_{{\hat{p}}_{j}}\left(Y_{i}-X_{i}^{T}{\hat{\beta }}_{j};0,{\hat{\sigma }}_{j}\right)\right\}+\log n*df,$$

where $\hat{m}$ denotes the estimate of the number of components, $df=3\hat{m}-1+{\hat{M}}_{\beta }$, and

$${\hat{M}}_{\beta }=\#\left\{\left|{\hat{\beta }}_{jt}\right|>10^{-3},j=1,\cdots ,\hat{m},t=1,\cdots ,p\right\}.$$

## 5. Simulation

In this section, we use some numerical simulations to illustrate the finite sample performance of the proposed method. For the penalty function, we use the SCAD penalty [22], which is given as follows:

$$p_{\lambda }\left(t;a\right)=\left\{\begin{array}{cc} \lambda |t|, & if\;|t|\leq \lambda ,\\ -(t^{2}-2a\lambda |t|+\lambda^{2})/\left[2\left(a-1\right)\right], & if\;\lambda <|t|\leq a\lambda ,\\ \left(a+1\right)\lambda^{2}/2, & otherwise, \end{array}\right.$$

where λ is a tuning parameter and a>2. According to the suggestion in Fan and Li [22], *a* is equal to 3.7 by minimizing the Bayes risks. The datasets are generated via a three-component FMLR model

(7) $$f\left(y|x\right)=\sum_{j=1}^{3}\pi_{j}f_{p_{j}}\left(y-x^{T}\beta_{j};0,\sigma_{j}\right),$$

where the components of x are generated independently from the 7-dimensional standard normal distribution. In detail, we generate random samples of each component from the following linear model

$$Y=X^{T}\beta +\epsilon .$$

We simulate 100 datasets from the FMLR model (7) with sample size of n=200, 600, 800, 1000. The datasets are generated by the following four scenarios:

**Scenario 1.** $\beta_{1}={\left(1,1,1,1,0,0,0\right)}^{T},\beta_{2}={\left(1,2,3,4,0,0,0\right)}^{T},\beta_{3}={\left(5,6,7,8,0,0,0\right)}^{T}$ and $\pi_{1}=0.4,\pi_{2}=0.3,\pi_{3}=0.3$, and the random error ϵ∼N(0,1);

**Scenario 2.** We use the same setting as in **Scenario 1**, except that the error term follows a t-distribution with freedom degree 2;

**Scenario 3.** We use the same setting as in **Scenario 1**, except that the error term follows a mixture t distribution: ϵ∼0.5t(1)+0.5t(3);

**Scenario 4.** We use the same setting as in **Scenario 1**, except that the error term follows a mixture normal distribution: $\epsilon ∼0.95N\left(0,1\right)+0.05N\left(0.5^{2}\right)$.

We compare our proposed method with the method proposed by [17]. To assess the finite-sample performance, we consider four different measures:

(1) $RMSE_{\pi_{j}}$: the root mean square error of $\hat{\pi_{j}}$ when the order is corrected estimated, which is defined by
  $$RMSE_{\pi_{j}}=\sqrt{\frac{1}{M^{*}}\sum_{m=1}^{M^{*}}{\left({\hat{\pi }}_{j}^{m}-\pi_{j}\right)}^{T}\left({\hat{\pi }}_{j}^{m}-\pi_{j}\right)}$$
  where $M^{*}$ is the number of simulations with correct estimation of the order.
(2) $RMSE_{\beta_{c}}$: the root mean square error of ${\hat{\beta }}_{j}$, which can be similarly calculated as $RMSE_{\pi_{j}}$.
(3) NCZ (the number of correct zeros): It denotes that the number of the true value of the parameter is zero and is correctly estimated as zero. NCZ can be calculated by
  $$NCZ=\#\left\{t:\beta_{t}=0\wedge {\hat{\beta }}_{t}=0\right\},$$
  where #{A} denotes the number of elements within A.
(4) NIZ (the number of incorrect zeros): It indicates that the number of the true value of the parameter is non-zero and is incorrectly estimated as zero. NIZ is given as follows:
  $$NIZ=\#\left\{t:\beta_{t}\neq 0\wedge {\hat{\beta }}_{t}=0\right\}.$$

In simulation studies, suppose we know that the data come from a mixture regression model with at most five components, but the true number of components should be estimated. For each scenario, the simulation is repeated 100 times. The corresponding results are shown in Table 1, Table 2, Table 3, Table 4, Table 5, Table 6, Table 7 and Table 8. In these Tables, M1 and M2 denote the results by [17] and our proposed method, respectively.

Table 1 shows the simulation results of order selection. Columns labeled “Underfitted” are the proportion of the fitted model with less than three components in 100 simulations. Meanwhile, “Correctly fitted” and “Overfitted” can be similarly interpreted. From Table 1, we can find that the effects of the two models are very similar, and the accuracy rate of order selection can reach more than 98% for M1 and M2 when *n* is larger than or equal to 600. Table 2 presents the results of variable selection and parameter estimation for each component. From Table 2, we observe that the finite sample performances of the two models are very similar for n≥600. Therefore, when the error term follows a normal distribution, the two models have similar performance when the sample size is sufficiently large.

Table 3 and Table 4 present the results of Scenario 2, which is a heavy-tailed scenario. We can observe from Table 3 that M1 can only estimate about 20% underfitted or overfitted model, while our method keeps robustness and continues to maintain 98% accuracy when n≥600. In Table 4, M1 has a poor performance in variable selection. M1 has many non-zero NIZ, while our method’s NIZ is all zero for n≥600. Meanwhile, the NCZ of our proposed method increases as *n* increases. In addition, our proposed method has a smaller RMSE than M1.

For Table 5, the performance of order selection for M1 is worse than that for M2. The ratio of the correctly fitted model remains above 98% with our method for n≥600, while M1 is easy to overfit the model’s components. In Table 6, it can be seen that the NCZ value of M1 is a little better than that of M2. Compared with $RMSE_{\beta_{c}}$, we can find that our method is better than M1 consistently.

Table 7 and Table 8 present the results of Scenario 4. M1 absolutely stays away from the right number of components. On the contrary, our method can select the correct number of components with 98% accuracy for n≥600. In Table 8, M1 is better than M2 in NCZ, but M1 is unstable in NIZ. Comparing $RMSE_{\beta_{c}}$, we can find that M1 is larger than M2. In general, our model is better than M1 in both order selection and variable selection and parameter estimation.

## 6. Real Data Analysis

In this section, we apply the proposed methodology to analyze baseball salary data, which consists of information about major league baseball players. The response variable is their 1992 salaries (measured in thousands of dollars). In addition, there are 16 performance measures for 337 MLB players who participated in at least one game in both the 1991 and 1992 seasons. This data set has been analyzed by others, such as [12,17]. We want to study how the performance measures affect salaries using our method.

The performance measures are batting average $\left(x_{1}\right)$, on-base percentage $\left(x_{2}\right)$, runs $\left(x_{3}\right)$, hits $\left(x_{4}\right)$, doubles $\left(x_{5}\right)$, triples $\left(x_{6}\right)$, home runs $\left(x_{7}\right)$, runs batted in $\left(x_{8}\right)$, walks $\left(x_{9}\right)$, strikeouts $\left(x_{10}\right)$, stolen bases $\left(x_{11}\right)$, and errors $\left(x_{12}\right)$; and indicators of free agency eligibility $\left(x_{13}\right)$, free agent in 1991/2 $\left(x_{14}\right)$, arbitration eligibility $\left(x_{15}\right)$, and arbitration in 1991/2 $\left(x_{16}\right)$. The four (dummy) variables $x_{13}-x_{16}$ indicate how free each player was to move to another team. As suggested in [25], the interaction effects between (dummy) variables $x_{13}-x_{16}$ and the quantitative variables $x_{1},x_{3},x_{7}$, and $x_{8}$ should be added to the consideration. Therefore, we obtain a set of 32 potential covariates affecting each player’s salary. Ref. [12] fitted a mixture of linear regression models with two or three components to depict the overlaid shape of the histogram of log(salary), and concluded that a two-component mixture regression model labeled MIXSCAD fitted the data well. [17] uses an FMLR model based on normal distribution, and the number of components is two.

As advocated by [12], we use log(salary) as the response variable. We first fit a linear model via stepwise regression, the results are shown in Table 9, denoted as ${\hat{\beta }}_{ols}$. Based on [17], we consider the following four-component mixture model,

$$Y|X∼\sum_{j=1}^{4}\pi_{j}f_{p_{j}}\left(Y-X^{T}\beta_{j};0,\sigma_{j}\right),$$

where Y = log(salary) and X is a 33×1 vector containing 32 covariates plus an intercept term. In order to implement the proposed modified EM algorithm, we set the initial values as follows

$$\pi^{0}={\left(0.4,0.2,0.2,0.2\right)}^{T},\sigma^{0}={\left(10,10,10,10\right)}^{T},p^{0}={\left(1,1,1,1\right)}^{T},\beta_{j}^{0}={\hat{\beta }}_{ols}+\epsilon_{j}$$

where $\epsilon_{j}∼N\left(0,I\right),j=1,2,3,4$. The results are reported in Table 9 and Table 10. From Table 9, we find that both M1 and M2 choose two components. Furthermore, we can observe from Table 10 that M2 has a smaller BIC value than M1, which indicates that our proposed method can better fit this dataset than M1.

Of interest is to explain how the performance measures affect salaries by interpreting the outcome of the fit, although it can be a source of controversy. Do not think about it; there should be many positive correlations between a baseball player and his salary. M1 and M2 have the same sign and approximate coefficients in $x_{0},x_{13},x_{15}$, and interactions of $x_{8}$ and $x_{14}$. Recall that $x_{1}$ and $x_{7}$ are individual performances, while $x_{13}$, $x_{15}$, and $x_{16}$ are three dummy variables indicating how freely players change teams. For example, the effect of $x_{1}*x_{16}$ implies that for most players having arbitration eligibility in 1991/2 enhances the individual ability $\left(x_{1}\right)$ toward a lower salary, but not the value of their team contribution $\left(x_{8}\right)$.

The main differences between the two models are interaction effects $x_{1}*x_{14}$ and $x_{1}*x_{15}$. This implies that M1 disregards $x_{1}*x_{14}$’s effect, but M2 indicates that it is in two directions. And M2 attaches great importance to the interaction effect of $x_{1}*x_{14}$.

## 7. Discussion

In this paper, we introduced the FMLR models via an exponential power distribution. Under some conditions, the asymptotic properties of the proposed estimators were established. Meanwhile, a modified EM algorithm and an MM algorithm were applied to solve the proposed optimization problem. Furthermore, the merits of our proposed methodology were illustrated through some numerical simulations and real data analysis. Simulation studies showed that the proposed method had better performance than the existing methods under difference errors. By analyzing a baseball salary dataset, our proposed method had a smaller BIC value than the method proposed [17].

## Figures and Tables

**Table 1:** Order selection results in Scenario 1.

n	M1			M2		
	Underfitted	Correctly Fitted	Overfitted	Underfitted	Correctly Fitted	Overfitted
200	0.00	0.99	0.01	0.40	0.60	0.00
600	0.00	0.99	0.01	0.00	0.99	0.01
800	0.00	0.99	0.01	0.00	0.99	0.01
1000	0.00	1.00	0.00	0.00	0.99	0.01

**Table 2:** Variable selection and parameter estimation results in Scenario 1.

n	M1				M2			
	${\mathrm{RMSE}}_{\pi_{c}}$	${\mathrm{RMSE}}_{\beta_{c}}$	NCZ	NIZ	${\mathrm{RMSE}}_{\pi_{c}}$	${\mathrm{RMSE}}_{\beta_{c}}$	NCZ	NIZ
200	0.092	0.535	2.900	0.200	0.143	0.352	2.667	0.000
	0.048	0.596	2.700	0.000	0.141	0.207	2.333	0.000
	0.073	0.703	2.670	0.000	0.048	0.427	2.833	0.000
600	0.024	0.154	2.990	0.000	0.023	0.156	2.990	0.000
	0.025	0.153	2.980	0.000	0.025	0.151	2.990	0.000
	0.021	0.154	2.990	0.000	0.022	0.156	2.980	0.000
800	0.022	0.142	2.990	0.000	0.020	0.145	3.000	0.000
	0.020	0.138	2.980	0.000	0.021	0.153	3.000	0.000
	0.019	0.141	2.990	0.000	0.020	0.138	3.000	0.000
1000	0.014	0.123	2.990	0.000	0.015	0.130	3.000	0.000
	0.016	0.122	3.000	0.000	0.014	0.121	3.000	0.000
	0.014	0.121	3.000	0.000	0.014	0.122	3.000	0.000

**Table 3:** Order selection results in Scenario 2.

n	M1			M2		
	Underfitted	Correctly Fitted	Overfitted	Underfitted	Correctly Fitted	Overfitted
200	0.50	0.20	0.30	0.16	0.64	0.20
600	0.07	0.81	0.12	0.00	0.99	0.01
800	0.03	0.75	0.22	0.01	0.98	0.01
1000	0.11	0.84	0.05	0.00	0.99	0.01

**Table 4:** Variable selection and parameter estimation results in Scenario 2.

n	M1				M2			
	${\mathrm{RMSE}}_{\pi_{c}}$	${\mathrm{RMSE}}_{\beta_{c}}$	NCZ	NIZ	${\mathrm{RMSE}}_{\pi_{c}}$	${\mathrm{RMSE}}_{\beta_{c}}$	NCZ	NIZ
200	0.285	8.381	2.500	0.000	0.088	0.964	2.722	0.056
	0.126	0.457	1.500	0.000	0.058	1.957	2.778	0.076
	0.223	2.876	2.000	2.000	0.090	0.852	2.772	0.000
600	0.057	0.671	2.893	0.000	0.048	0.261	2.963	0.000
	0.062	1.119	2.844	0.011	0.040	0.240	2.876	0.000
	0.055	1.264	2.872	0.034	0.044	0.264	2.896	0.000
800	0.065	0.715	2.897	0.013	0.034	0.223	2.845	0.000
	0.053	0.874	2.892	0.012	0.032	0.228	2.957	0.000
	0.047	1.241	2.887	0.000	0.033	0.193	2.929	0.000
1000	0.063	0.905	2.912	0.012	0.029	0.198	2.906	0.000
	0.056	0.926	2.923	0.011	0.031	0.191	2.946	0.000
	0.047	0.837	2.921	0.012	0.033	0.188	2.979	0.000

**Table 5:** Order selection results in Scenario 3.

n	M1			M2		
	Underfitted	Correctly Fitted	Overfitted	Underfitted	Correctly Fitted	Overfitted
200	0.60	0.25	0.15	0.32	0.66	0.02
600	0.00	0.74	0.26	0.00	0.98	0.02
800	0.03	0.73	0.24	0.00	0.99	0.01
1000	0.05	0.79	0.16	0.00	0.99	0.01

**Table 6:** Variable selection and parameter estimation results in Scenario 3.

n	M1				M2			
	${\mathrm{RMSE}}_{\pi_{c}}$	${\mathrm{RMSE}}_{\beta_{c}}$	NCZ	NIZ	${\mathrm{RMSE}}_{\pi_{c}}$	${\mathrm{RMSE}}_{\beta_{c}}$	NCZ	NIZ
200	0.236	2.312	2.000	0.000	0.039	0.766	3.000	0.500
	0.165	3.903	2.400	0.200	0.054	0.969	3.000	0.167
	0.060	1.732	2.600	1.400	0.049	0.929	3.000	0.667
600	0.025	0.164	2.887	0.000	0.023	0.122	2.874	0.000
	0.025	0.156	2.889	0.000	0.024	0.127	2.869	0.000
	0.027	0.162	2.896	0.000	0.025	0.124	2.877	0.000
800	0.024	0.154	2.893	0.000	0.018	0.114	2.878	0.000
	0.023	0.137	2.886	0.000	0.017	0.123	2.931	0.000
	0.019	0.134	2.897	0.000	0.017	0.123	2.931	0.000
1000	0.021	0.132	2.894	0.000	0.017	0.097	2.924	0.000
	0.020	0.138	2.924	0.000	0.017	0.097	2.924	0.000
	0.019	0.122	2.891	0.000	0.016	0.114	2.971	0.000

**Table 7:** Order selection results in Scenario 4.

n	M1			M2		
	Underfitted	Correctly Fitted	Overfitted	Underfitted	Correctly Fitted	Overfitted
200	0.49	0.41	0.10	0.07	0.72	0.21
600	0.13	0.28	0.59	0.02	0.98	0.00
800	0.19	0.31	0.50	0.00	1.00	0.00
1000	0.10	0.39	0.51	0.01	0.99	0.00

**Table 8:** Variable selection and parameter estimation results in Scenario 4.

n	M1				M2			
	${\mathrm{RMSE}}_{\pi_{c}}$	${\mathrm{RMSE}}_{\beta_{c}}$	NCZ	NIZ	${\mathrm{RMSE}}_{\pi_{c}}$	${\mathrm{RMSE}}_{\beta_{c}}$	NCZ	NIZ
200	0.014	5.455	2.889	0.444	0.218	0.971	2.500	0.000
	0.180	1.337	2.889	0.222	0.228	1.669	2.000	0.000
	0.079	2.956	2.889	0.000	0.320	2.284	2.250	0.000
600	0.050	2.672	2.832	0.000	0.054	0.372	2.776	0.000
	0.053	1.256	2.717	0.000	0.055	0.273	2.724	0.000
	0.054	2.136	2.846	0.038	0.061	0.271	2.878	0.000
800	0.051	1.134	2.811	0.000	0.035	0.398	2.600	0.000
	0.045	1.535	2.623	0.000	0.039	0.163	2.600	0.000
	0.047	2.724	2.747	0.000	0.022	0.183	3.000	0.000
1000	0.074	1.217	2.942	0.000	0.035	0.347	2.973	0.000
	0.073	1.736	2.974	0.103	0.031	0.220	2.697	0.000
	0.047	3.734	2.772	0.000	0.025	0.129	2.949	0.000

**Table 9:** Parameter estimates for baseball salary data.

Covariates	Linear Model	M1		M2	
		Comp1	Comp2	Comp1	Comp2
$x_{0}$	5.48	4.81	5.66	4.70	4.67
$x_{1}$	-	-	-	-	-
$x_{2}$	−1.54	-	-	-	-
$x_{3}$	-	-	-	-	-
$x_{4}$	-	-	0.01	0.03	0.02
$x_{5}$	-	-	-	-	0.01
$x_{6}$	-	-	-	-	-
$x_{7}$	-	-	-	-	-
$x_{8}$	0.01	0.01	0.02	0.01	-
$x_{9}$	0.01	-	-	0.03	0.01
$x_{10}$	−0.01	-	-	-	-
$x_{11}$	-	0.03	-	-	0.01
$x_{12}$	-	-	-	-	-
$x_{13}$	1.52	2.04	-	3.13	2.16
$x_{14}$	-0.48	-	-	-	-
$x_{15}$	1.35	1.60	-	2.73	1.28
$x_{16}$	-	-	-	0.01	1.40
$x_{1}*x_{13}$	-	-	-	-	-
$x_{1}*x_{14}$	-	-	-	-	10.05
$x_{1}*x_{15}$	-	-	-	0.01	-
$x_{1}*x_{16}$	−4.38	-	-	-	-
$x_{3}*x_{13}$	-	-	-	-	-
$x_{3}*x_{14}$	-	-	-	−0.01	−0.02
$x_{3}*x_{15}$	-	-	-	-	-
$x_{3}*x_{16}$	-	-	-	0.01	-
$x_{7}*x_{13}$	0.01	-	-	0.03	-
$x_{7}*x_{14}$	0.03	-	-	-	0.02
$x_{7}*x_{15}$	-	-	-	-	-
$x_{7}*x_{16}$	-	-	-	-	-
$x_{8}*x_{13}$	-	-	0.01	-	0.01
$x_{8}*x_{14}$	-	0.01	-	0.01	-
$x_{8}*x_{15}$	-	-	0.02	-	-
$x_{8}*x_{16}$	0.02	-	-	-	0.02

**Table 10:** Model parameter estimates for baseball salary data.

Parameter	M1		M2	
$\hat{\pi }$	0.69	0.31	0.84	0.16
$\hat{p}$	2	2	1.05	1.49
$\hat{\sigma }$	-	-	2.27	7.13
$\lambda_{1}$	0.300		0.220	
$\lambda_{2}$	0.040		0.016	
MBIC	569.64		**547.25**	

## Data Availability

The data used in this study is publicly available. Code is available on request from the second authors.

## Appendix A

**Proof** **of Theorem 1.** For any given ϵ>0, let $∥u∥=M_{\epsilon }$. Denote

$$\Gamma_{n}\left(u\right)={\tilde{Q}}_{n}\left(\theta_{0}+u/\sqrt{n}\right)-{\tilde{Q}}_{n}\left(\theta_{0}\right).$$

According to (2), we have

$$\Gamma_{n}\left(u\right)=\left[Q_{n}\left(\theta_{0}+u/\sqrt{n}\right)-Q_{n}\left(\theta_{0}\right)\right]-\left[P_{n}^{1}\left(\theta_{0}+u/\sqrt{n}\right)-P_{n}^{1}\left(\theta_{0}\right)\right]-\left[P_{n}^{2}\left(\theta_{0}+u/\sqrt{n}\right)-P_{n}^{2}\left(\theta_{0}\right)\right].$$

Under condition (C1), we have $p_{\lambda }\left(0\right)=0$ for any λ. Therefore, $P_{n}^{1}\left(\theta_{0}\right)=P_{n}^{1}\left(\theta_{01}\right)$ and $P_{n}^{2}\left(\theta_{0}\right)=P_{n}^{2}\left(\theta_{01}\right)$. Since $P_{n}^{1}\left(\theta_{0}+u/\sqrt{n}\right)$ and $P_{n}^{2}\left(\theta_{0}+u/\sqrt{n}\right)$ are a sum of positive terms, we then have

$$\Gamma_{n}\left(u\right)\leq \left[Q_{n}\left(\theta_{0}+u/\sqrt{n}\right)-Q_{n}\left(\theta_{0}\right)\right]-\left[P_{n}^{1}\left(\theta_{01}+u_{1}/\sqrt{n}\right)-P_{n}^{1}\left(\theta_{01}\right)\right]-\left[P_{n}^{2}\left(\theta_{01}+u_{1}/\sqrt{n}\right)-P_{n}^{2}\left(\theta_{01}\right)\right],$$

where $u_{1}$ is a subvector of u with the corresponding non-zero coefficients. By conditions (C4), (C5), (C7) and (C8), and the Taylor’s expansion, we have

$$\begin{array}{c} Q_{n}\left(\theta_{0}+u/\sqrt{n}\right)-Q_{n}\left(\theta_{0}\right)=n^{-1/2}Q_{n}^{\prime }{\left(\theta_{0}\right)}^{T}u-\frac{1}{2}\left(u^{T}Q\left(\theta_{0}\right)u\right)\left(1+o_{p}\left(1\right)\right). \end{array}$$

By condition (C1), the Taylor’s expansion, triangular inequality, and Cauchy–Schwarz inequality, we have

$$\begin{array}{ccc} \; & P_{n}^{1}\left(\theta_{01}+u_{1}/\sqrt{n}\right)-P_{n}^{1}\left(\theta_{01}\right) & \\ \; & =n\sum_{k=1}^{m_{0}}\left\{\sum_{j=1}^{t_{k}}\left[p_{\lambda_{1}}\left(\right|\beta_{kj}+u_{kj}/\sqrt{n}\left|\right)-p_{\lambda_{1}}\left(\right|u_{kj}/\sqrt{n}\left|\right)\right]\right\} & \\ \; & =\sqrt{m_{0}t}a_{n}∥u∥+\frac{b_{n}}{2}{∥u∥}^{2}\left(1+o\left(1\right)\right), \end{array}$$

where $m_{0}$ is the number of true non-zero mixing weights, and $t=\max_{k}\sqrt{t_{k}}$, and $t_{k}$ is the number of true non-zero regression coefficients in the *k*-th component. Since $\sqrt{n}\lambda_{2}\to \infty$, and $\lambda_{2}\to 0$, we have

$$\begin{array}{cc} \; & |P_{n}^{2}\left(\theta_{01}+u_{1}/\sqrt{n}\right)-P_{n}^{2}\left(\theta_{01}\right)|=0. \end{array}$$

Regularity condition (C6) implies that $n^{-1/2}Q_{n}^{\prime }\left(\theta_{0}\right)=O_{p}\left(1\right)$. Since $\sqrt{n}\min\left\{\lambda_{1},\lambda_{2}\right\}\to \infty$, and $\min\{\lambda_{1},\lambda_{2}\}\to 0$, we have $a_{n}=0$. By conditions (C2) and (C6), for any given ϵ>0, there exists a sufficiently large $M_{\epsilon }$ such that

$$\begin{array}{c} \lim_{n\to \infty }P\left\{\underset{∥u∥=M_{\epsilon }}{\sup}\Gamma_{n}\left(u\right)<0\right\}\geq 1-\epsilon . \end{array}$$

Therefore, with large probability, there is a local maximum in $\left\{\theta +u/\sqrt{n}:∥u∥\leq M_{\epsilon }\right\}$. That is to say, this local maximizer ${\hat{\theta }}_{n}$ satisfies $∥{\hat{\theta }}_{n}-\theta_{0}∥=O_{p}\left(1/\sqrt{n}\right)$. This completes the proof of Theorem 1. □

**Proof** **of Theorem 2.** We first show that ${\hat{\pi }}_{k}=0$ for $k=m_{0}+1,\cdots ,m$. Since $\left|\left|{\hat{\theta }}_{n}-\theta \right|\right|=O_{p}\left(n^{-1/2}\right)$, we have ${\hat{\pi }}_{k}=O_{p}\left(1/\sqrt{n}\right)$ for $k=m_{0}+1,\cdots ,m$. To prove (a), it is sufficient to show with probability tending to 1 as n→∞ for any $\pi_{k}$ satisfying ${\hat{\pi }}_{k}-\pi_{k}=O_{p}\left(1/\sqrt{n}\right)$ and $k=m_{0}+1,\cdots ,m$

(A1) $$\begin{array}{c} \frac{\partial Q^{*}\left(\theta \right)}{\partial {\hat{\pi }}_{k}}<0\;for\;{\hat{\pi }}_{k}<C/\sqrt{n}, \end{array}$$

where *C* is a positive constant number,

$$\begin{array}{c} Q^{*}\left(\theta \right)={\tilde{Q}}_{n}\left(\theta \right)-\delta \left(\sum_{k=1}^{m}\pi_{k}-1\right), \end{array}$$

and δ is a Lagrange multiplier. Therefore, ${\hat{\pi }}_{k},k=1,\cdots ,m$ should satisfy

(A2) $$\begin{array}{c} \frac{\partial Q^{*}\left(\theta \right)}{\partial {\hat{\pi }}_{k}}=\sum_{i=1}^{n}\frac{f_{p_{j}}\left(Y_{i}-X_{i}^{T}\beta_{j};0,\sigma_{j}\right)}{\sum_{j=1}^{m}{\hat{\pi }}_{j}f_{p_{j}}\left(Y_{i}-X_{i}^{T}\beta_{j};0,\sigma_{j}\right)}-n\lambda_{2}\frac{p_{\lambda_{2}}^{\prime }\left({\hat{\pi }}_{k}\right)}{C_{0}+p_{\lambda_{2}}\left({\hat{\pi }}_{k}\right)}-\delta =0. \end{array}$$

We first consider $k\leq m_{0}$. By the law of large numbers, we have

(A3) $$\begin{array}{c} \sum_{i=1}^{n}\frac{f_{p_{j}}\left(Y_{i}-X_{i}^{T}\beta_{j};0,\sigma_{j}\right)}{\sum_{j=1}^{m}{\hat{\pi }}_{j}f_{p_{j}}\left(Y_{i}-X_{i}^{T}\beta_{j};0,\sigma_{j}\right)}=O_{p}\left(n\right). \end{array}$$

For $k\leq m_{0}$, we have ${\hat{\pi }}_{k}=\pi_{k}^{0}+O_{p}\left(1/\sqrt{n}\right)>\frac{1}{2}\min\left\{\pi_{1}^{0},\cdots ,\pi_{m_{0}}^{0}\right\}$. Since $n\lambda_{2}=o_{p}\left(n\right)$, $p_{\lambda_{2}}^{\prime }\left({\hat{\pi }}_{k}\right)=o_{p}\left(1\right)$ and $p_{\lambda_{2}}\left({\hat{\pi }}_{k}\right)=o_{p}\left(n\right)$, then we have

(A4) $$\begin{array}{c} n\lambda_{2}\frac{p_{\lambda_{2}}^{\prime }\left({\hat{\pi }}_{k}\right)}{C_{0}+p_{\lambda_{2}}\left({\hat{\pi }}_{k}\right)}=o_{p}\left(1\right). \end{array}$$

By (A2)–(A4), we have $\delta =O_{p}\left(n\right)$. For $k\geq m_{0}+1$ and ${\hat{\pi }}_{k}<C/\sqrt{n}$, we have ${\hat{\pi }}_{k}=O_{p}\left(1/\sqrt{n}\right)$. By $\sqrt{n}\lambda_{2}\to \infty$, $C_{0}$ is sufficient small and $p_{\lambda }\left(\cdot \right)$ is the SCAD penalty, we have

$$\begin{array}{c} \left\{n\lambda_{2}\frac{p_{\lambda_{2}}^{\prime }\left({\hat{\pi }}_{k}\right)}{C_{0}+p_{\lambda_{2}}\left({\hat{\pi }}_{k}\right)}\right\}/n=\frac{\lambda_{2}^{2}}{C_{0}+\lambda_{2}{\hat{\pi }}_{k}}=O_{p}\left(\sqrt{n}\lambda_{2}\right)\to \infty . \end{array}$$

Therefore, the first term and the third term in the Equation (A2) are dominated by the second term. Thus, we prove the Equation (A1). This completes the proof of (a). To prove (b), for any θ with $m_{0}$ components, we split $\theta_{m_{0}}=\left(\theta_{m_{0}}^{1},\theta_{m_{0}}^{2}\right)$ for any $\theta_{m_{0}}$ in the neighborhood $\left|\right|\theta_{m_{0}}-\theta_{m_{0}}^{0}\left|\right|=O_{p}\left(1/\sqrt{n}\right)$ such that $\theta_{m_{0}}^{2}$ contains all zero effects, e.g., $\beta_{kj}=0,k=1,\cdots ,m_{0}$ and $j=1,\cdots ,t_{k}$. By (2), we have

$$\begin{array}{cc} \; & {\tilde{Q}}_{n}\left\{\left(\theta_{m_{0}}^{1},\theta_{m_{0}}^{2}\right)\right\}-{\tilde{Q}}_{n}\left\{\left(\theta_{m_{0}}^{1},0\right)\right\}\\ \; & =\left[Q_{n}\left\{\left(\theta_{m_{0}}^{1},\theta_{m_{0}}^{2}\right)\right\}-Q_{n}\left\{\left(\theta_{m_{0}}^{1},0\right)\right\}\right]-\left[P_{n}^{1}\left\{\left(\theta_{m_{0}}^{1},\theta_{m_{0}}^{2}\right)\right\}-P_{n}^{1}\left\{\left(\theta_{m_{0}}^{1},0\right)\right\}\right]\\ \; & =\left[Q_{n}\left\{\left(\theta_{m_{0}}^{1},\theta_{m_{0}}^{2}\right)\right\}-Q_{n}\left\{\left(\theta_{m_{0}}^{1},0\right)\right\}\right]-n\sum_{k=1}^{m_{0}}\sum_{j=t_{k}+1}^{p}p_{\lambda_{1}}\left(\right|\beta_{kj}\left|\right). \end{array}$$

According to the mean value theorem, we have

(A5) $$\begin{array}{c} Q_{n}\left\{\left(\theta_{m_{0}}^{1},\theta_{m_{0}}^{2}\right)\right\}-Q_{n}\left\{\left(\theta_{m_{0}}^{1},0\right)\right\}={\left[\frac{\partial Q_{n}\left\{\left(\theta_{m_{0}}^{1},\gamma \right)\right\}}{\partial \theta_{m_{0}}^{2}}\right]}^{T}\theta_{m_{0}}^{2}, \end{array}$$

where $\left||\gamma |\right|\leq \left|\right|\theta_{m_{0}}^{2}\left|\right|=O\left(n^{-1/2}\right)$. Since

$$\begin{array}{cc} \; & \left|\left|\frac{\partial Q_{n}\left\{\left(\theta_{m_{0}}^{1},\gamma \right)\right\}}{\partial \theta_{m_{0}}^{2}}-\frac{\partial Q_{n}\left\{\left(\theta_{m_{0}}^{01},0\right)\right\}}{\partial \theta_{m_{0}}^{2}}\right|\right|\\ \; & \leq \left|\left|\frac{\partial Q_{n}\left\{\left(\theta_{m_{0}}^{1},\gamma \right)\right\}}{\partial \theta_{m_{0}}^{2}}-\frac{\partial Q_{n}\left\{\left(\theta_{m_{0}}^{1},0\right)\right\}}{\partial \theta_{m_{0}}^{2}}\right|\right|+\left|\left|\frac{\partial Q_{n}\left\{\left(\theta_{m_{0}}^{1},0\right)\right\}}{\partial \theta_{m_{0}}^{2}}-\frac{\partial Q_{n}\left\{\left(\theta_{m_{0}}^{01},0\right)\right\}}{\partial \theta_{m_{0}}^{2}}\right|\right|\\ \; & \leq \left[\sum_{i=1}^{n}R_{1}\left(z_{i}\right)\right]\left||\gamma |\right|+\left[\sum_{i=1}^{n}R_{1}\left(z_{i}\right)\right]\left|\right|\theta_{m_{0}}^{1}-\theta_{m_{0}}^{01}\left|\right|\\ \; & =\left(||\gamma |\right|+\left|\right|\theta_{m_{0}}^{1}-\theta_{m_{0}}^{01}\left||\right)O_{p}\left(n\right)=O_{p}\left(n^{1/2}\right), \end{array}$$

and

$$\begin{array}{c} \frac{\partial Q_{n}\left\{\left(\theta_{m_{0}}^{01},0\right)\right\}}{\partial \theta_{m_{0}}^{2}}=O_{p}\left(n^{1/2}\right), \end{array}$$

we have

(A6) $$\begin{array}{c} \frac{\partial Q_{n}\left\{\left(\theta_{m_{0}}^{1},\gamma \right)\right\}}{\partial \theta_{m_{0}}^{2}}=O_{p}\left(n^{1/2}\right). \end{array}$$

By (A5) and (A6), we have

$$\begin{array}{c} Q_{n}\left\{\left(\theta_{m_{0}}^{1},\theta_{m_{0}}^{2}\right)\right\}-Q_{n}\left\{\left(\theta_{m_{0}}^{1},0\right)\right\}=O_{p}\left(n^{1/2}\right)\left[\sum_{k=1}^{m_{0}}\sum_{j=t_{k}+1}^{p}\left|\beta_{kj}\right|\right]. \end{array}$$

Thus, we have

$$\begin{array}{c} {\tilde{Q}}_{n}\left\{\left(\theta_{m_{0}}^{1},\theta_{m_{0}}^{2}\right)\right\}-{\tilde{Q}}_{n}\left\{\left(\theta_{m_{0}}^{1},0\right)\right\}=\sum_{k=1}^{m_{0}}\sum_{j=t_{k}+1}^{p}\left[O_{p}\left(n^{1/2}\right)\left|\beta_{kj}\right|-np_{\lambda_{1}}\left(\right|\beta_{kj}\left|\right)\right]. \end{array}$$

By condition (C3), for $\left|t\right|\leq n^{-1/2}\log n$, we have $O_{p}\left(n^{1/2}\right)|t|<np_{\lambda_{1}}\left(|t|\right)$. Therefore, we can obtain

(A7) $$\begin{array}{c} {\tilde{Q}}_{n}\left\{\left(\theta_{m_{0}}^{1},\theta_{m_{0}}^{2}\right)\right\}-{\tilde{Q}}_{n}\left\{\left(\theta_{m_{0}}^{1},0\right)\right\}<0. \end{array}$$

By (A7), with probability tending to 1 as n→∞, we have

$$\begin{array}{cc} \; & {\tilde{Q}}_{n}\left\{\left(\theta_{m_{0}}^{1},\theta_{m_{0}}^{2}\right)\right\}-{\tilde{Q}}_{n}\left\{\left({\hat{\theta }}_{m_{0}}^{1},0\right)\right\}\\ \; & =\left[{\tilde{Q}}_{n}\left\{\left(\theta_{m_{0}}^{1},\theta_{m_{0}}^{2}\right)\right\}-{\tilde{Q}}_{n}\left\{\left(\theta_{m_{0}}^{1},0\right)\right\}\right]+\left[{\tilde{Q}}_{n}\left\{\left(\theta_{m_{0}}^{1},0\right)\right\}-{\tilde{Q}}_{n}\{\left({\hat{\theta }}_{m_{0}}^{1},0)\right\}\right]<0. \end{array}$$

Thus, this completes the proof of part (b). Using the result of Theorem 1, there exists a $\sqrt{n}$-consistent local maximizer ${\hat{\theta }}_{n1}$ of ${\tilde{Q}}_{n}\left\{\left(\theta_{1},0\right)\right\}$ such that ${\hat{\theta }}_{n}=\left({\hat{\theta }}_{n1},0\right)$ satisfies

(A8) $$\frac{\partial {\tilde{Q}}_{n}\left({\hat{\theta }}_{n}\right)}{\partial \theta_{1}}={\left\{\frac{Q_{n}\left(\theta \right)}{\partial \theta_{1}}-\frac{\partial P_{n}^{1}\left(\theta \right)}{\partial \theta_{1}}-\frac{\partial P_{n}^{2}\left(\theta \right)}{\partial \theta_{1}}\right\}}_{\theta ={\hat{\theta }}_{n}}=0.$$

By the Taylor’s expansion, we have

(A9) $${\left\{\frac{\partial Q_{n}\left(\theta \right)}{\partial \theta_{1}}\right\}}_{\theta ={\hat{\theta }}_{n}}=\frac{\partial Q_{n}\left(\theta_{01}\right)}{\partial \theta_{1}}+\left\{\frac{\partial^{2}Q_{n}\left(\theta_{01}\right)}{\partial \theta_{1}\partial \theta_{1}^{T}}+o_{p}\left(n\right)\right\}\left({\hat{\theta }}_{n1}-\theta_{01}\right),$$

(A10) $${\left\{\frac{\partial P_{n}^{1}\left(\theta \right)}{\partial \theta_{1}}\right\}}_{\theta ={\hat{\theta }}_{n}}=P_{n}^{1^{\prime }}\left(\theta_{01}\right)+\left\{P_{n}^{1^{\prime \prime }}\left(\theta_{01}\right)+o_{p}\left(n\right)\right\}\left({\hat{\theta }}_{n1}-\theta_{01}\right),$$

(A11) $${\left\{\frac{\partial P_{n}^{2}\left(\theta \right)}{\partial \theta_{1}}\right\}}_{\theta ={\hat{\theta }}_{n}}=P_{n}^{2^{\prime }}\left(\theta_{01}\right)+\left\{P_{n}^{2^{\prime \prime }}\left(\theta_{01}\right)+o_{p}\left(n\right)\right\}\left({\hat{\theta }}_{n1}-\theta_{01}\right).$$

By substituting Equations (A9)–(A11) into (A8), we have

$$\begin{array}{cc} \; & \left\{\frac{\partial^{2}Q_{n}\left(\theta_{01}\right)}{\partial \theta_{1}\partial \theta_{1}^{T}}-P_{n}^{1^{\prime \prime }}\left(\theta_{01}\right)-P_{n}^{2^{\prime \prime }}\left(\theta_{01}\right)+o_{p}\left(n\right)\right\}\left({\hat{\theta }}_{n1}-\theta_{01}\right)\\ \; & =\frac{\partial Q_{n}\left(\theta_{01}\right)}{\partial \theta_{1}}-P_{n}^{1^{\prime }}\left(\theta_{01}\right)-P_{n}^{2^{\prime }}\left(\theta_{01}\right). \end{array}$$

By the conditions (C4), (C5), and (C6), we have

$$\begin{array}{c} \frac{1}{n}\frac{\partial^{2}Q_{n}\left(\theta_{01}\right)}{\partial \theta_{1}\partial \theta_{1}^{T}}=Q_{1}\left(\theta_{01}\right)+o_{p}\left(1\right), \end{array}$$

$$\begin{array}{c} \frac{1}{\sqrt{n}}\frac{\partial Q_{n}\left(\theta_{01}\right)}{\partial \theta_{1}}\overset{D}{\to }N\left(0,Q_{1}\left(\theta_{01}\right)\right). \end{array}$$

By Slutsky’s theorem, we have

$$\begin{array}{c} \sqrt{n}\left\{\left[Q_{1}\left(\theta_{01}\right)-\frac{P_{n}^{1^{\prime \prime }}\left(\theta_{01}\right)}{n}-\frac{P_{n}^{2^{\prime \prime }}\left(\theta_{01}\right)}{n}\right]\left({\hat{\theta }}_{n1}-\theta_{01}\right)+\frac{P_{n}^{1^{\prime }}\left(\theta_{01}\right)}{n}+\frac{P_{n}^{2^{\prime }}\left(\theta_{01}\right)}{n}\right\}\overset{D}{\to }N\left(0,Q_{1}\left(\theta_{01}\right)\right). \end{array}$$

This completes the proof of part (c). □
